# Supplementary material for: Inhibitors of the PqsR Quorum-Sensing Receptor Reveal Differential Roles for PqsE and RhlI in Control of Phenazine Production
Source: ACS Chem Biol. 2025 May 14;20(6):1273–87. doi: 10.1021/acschembio.5c00114 (PMC12186256; doi:10.1021/acschembio.5c00114)
Supplement: Supplementary file 1 [file cb5c00114_si_001.pdf]

Supporting Information

Inhibitors of the PqsR Quorum-Sensing Receptor Reveal Differential Roles for PqsE and RhlI in Control of Phenazine Production

Julie S. Valastyan[1,2], Emilee E. Shine[1], Robert A. Mook[3], and Bonnie L. Bassler[1,2]

1. Department of Molecular Biology, Princeton University, Princeton, New Jersey 08544, United States.
2. Howard Hughes Medical Institute, Chevy Chase, Maryland 20815, United States.
3. Department of Medicine, Duke University Medical Center, Durham, North Carolina 27710, United States.

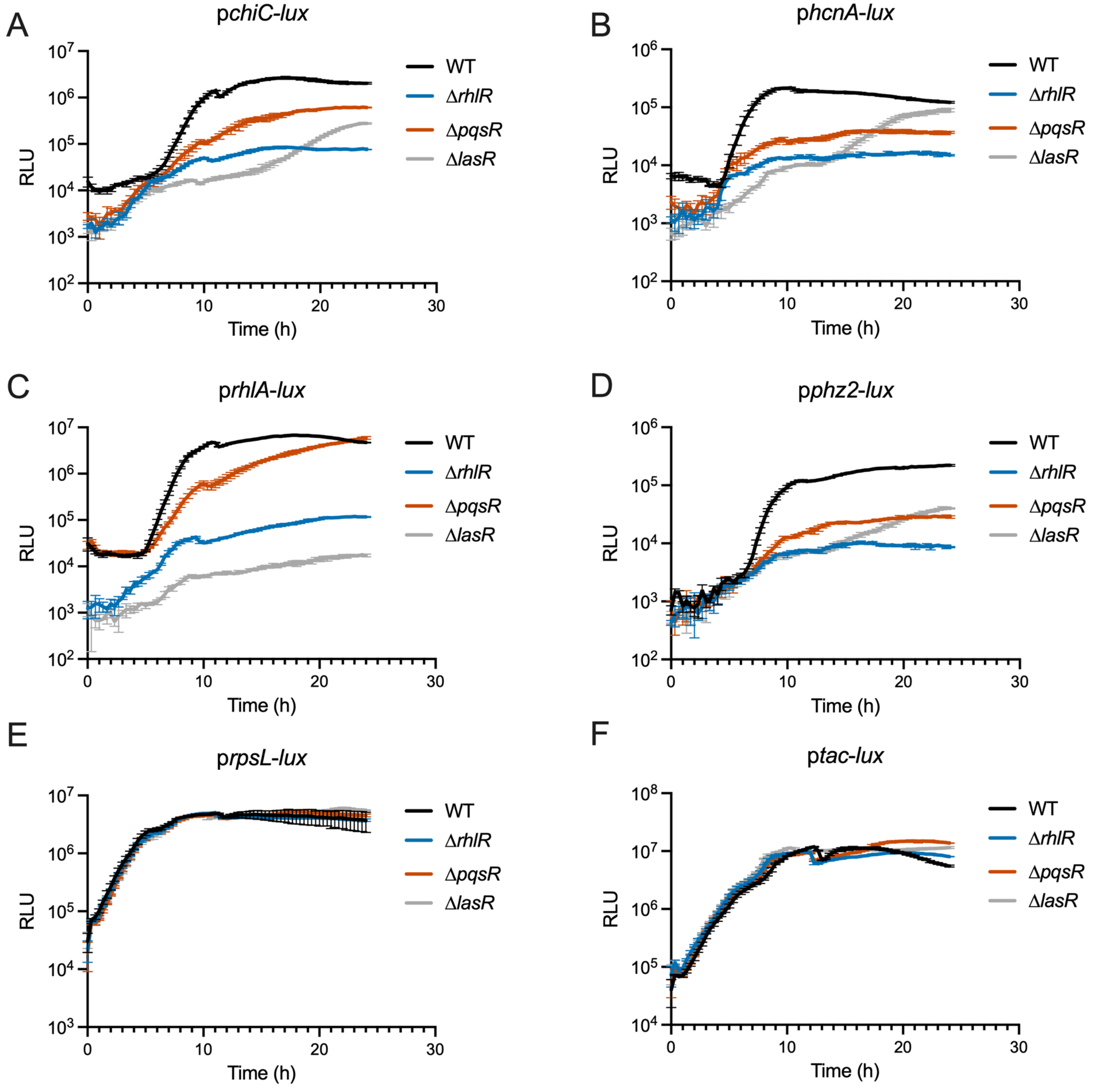

Figure S1. Light production over time in the designated *P. aeruginosa* strains carrying (A) p*chiC*-*lux*, (B) p*hcnA*-*lux*, (C) p*rhlA*-*lux*, (D) p*phz2*-*lux*, (E) p*rpsL*-*lux*, and (F) p*tac*-*lux*. RLU as in Figure 1. In all panels, error bars = standard deviations of biological replicates, $n = 3$.

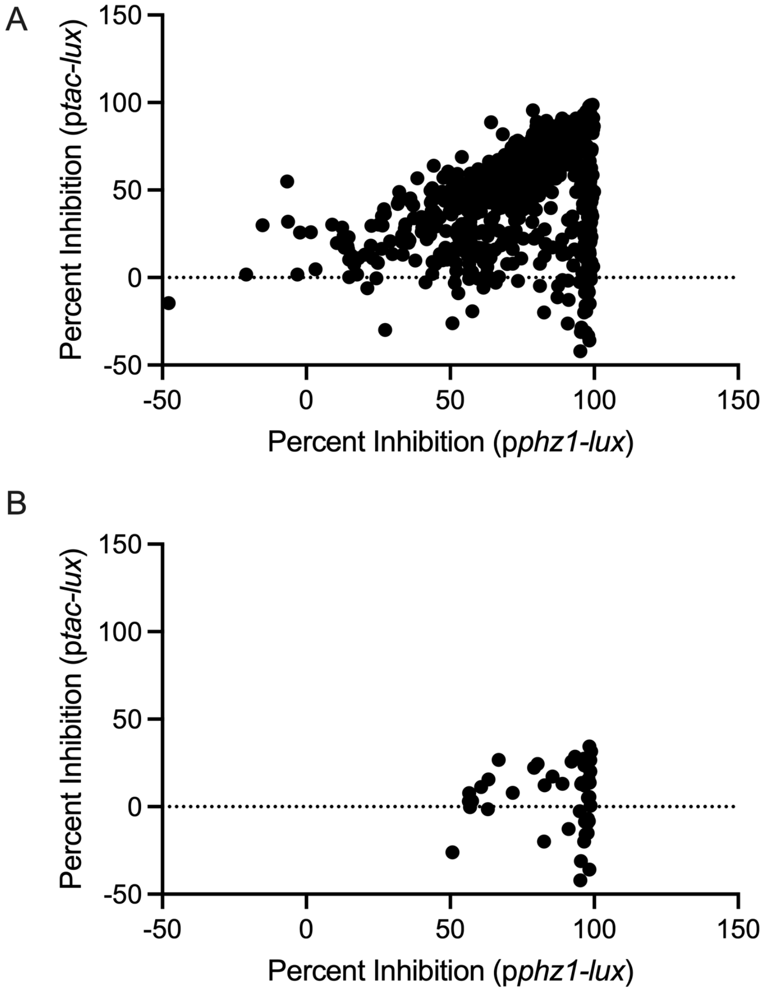

Figure S2. Percent inhibition of light production from the p*tac-lux* reporter versus percent inhibition of light production from the p*phz1-lux* reporter in WT *P. aeruginosa* for (A) all compounds tested in step 3 of the screen and (B) the final compounds from step 5 of the screen.

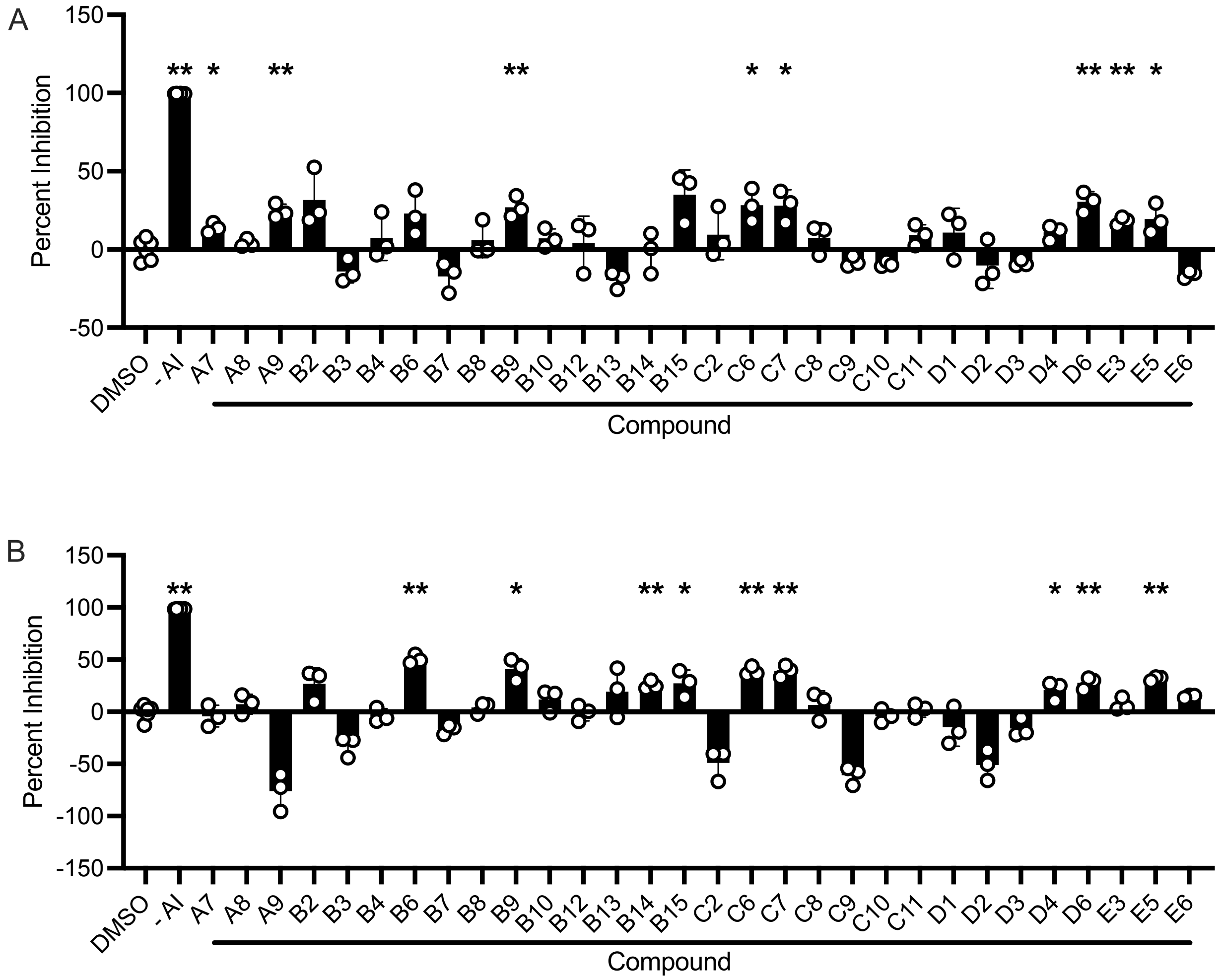

Figure S3. Inhibition of light production from the (A) p*rhlA-lux* and (B) p*lasB-lux* reporters in *E. coli* by 100 µM of the designated candidate compounds. The second bar in each panel shows the results when no C4-HSL (panel A) or no 3O-C12-HSL (panel B) autoinducer was added. For all other bars, either 10 µM C4-HSL (panel A) or 5 nM 3O-C12-HSL (panel B) was present. Error bars = standard deviations of biological replicates, $n = 3$, * = $p < 0.05$ and ** = $p < 0.005$ in two-tailed Student's t-test.

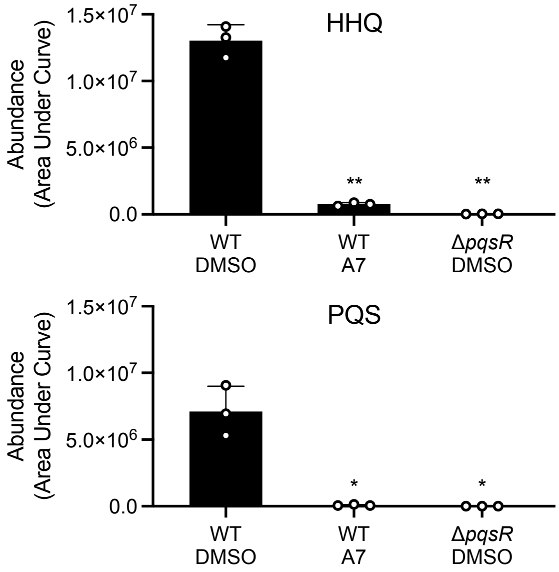

Figure S4. Quantitation of HHQ and PQS production in WT *P. aeruginosa* following treatment with DMSO or 100 μM of compound A7 compared that that produced by Δ*pqsR P. aeruginosa*. Error bars = standard deviations of biological replicates, $n = 3$, * = $p < 0.05$ and ** = $p < 0.005$ in two-tailed Student's t-test.

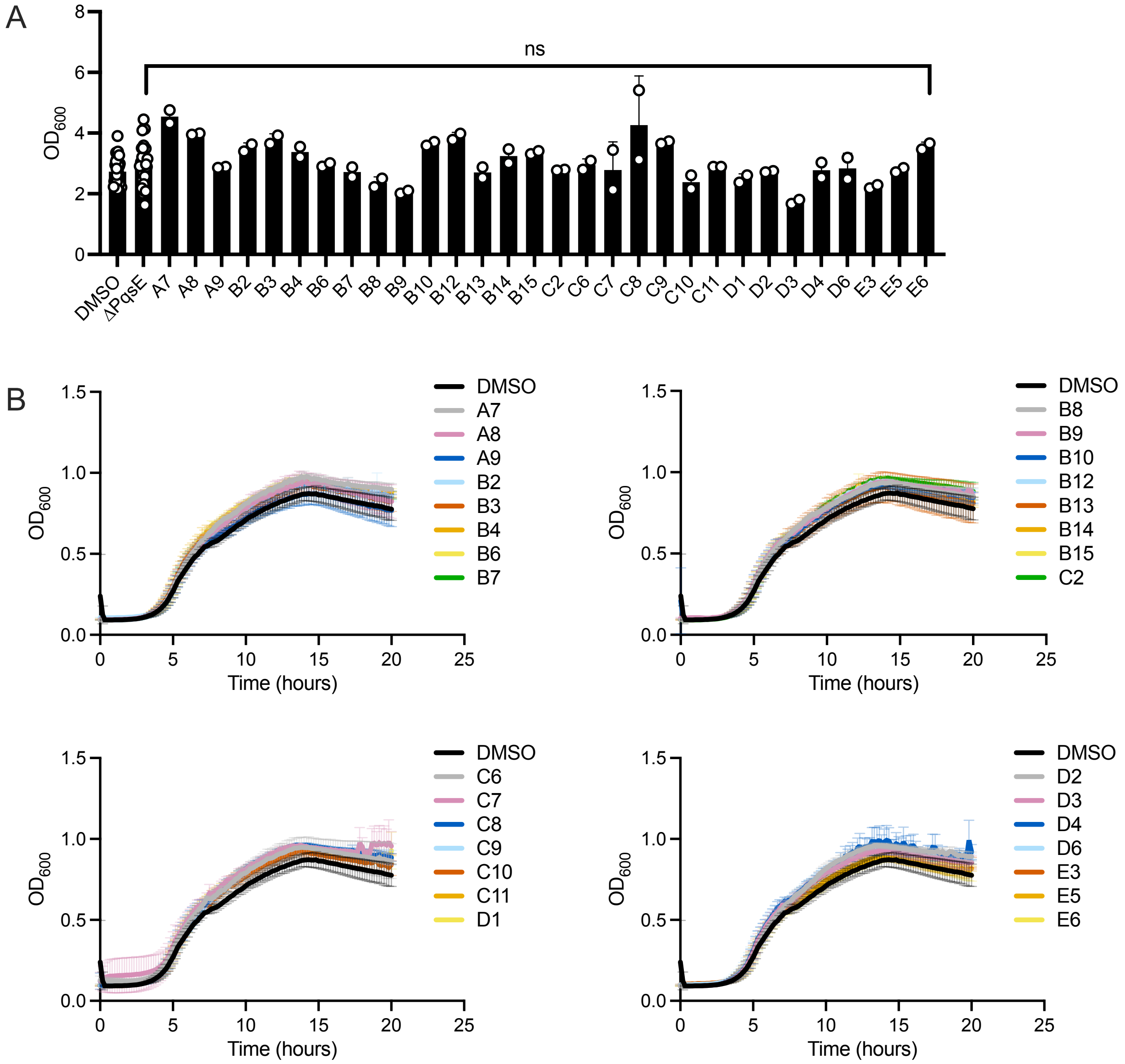

Figure S5. (A) Final OD_{600} readings for cultures used in the pyocyanin assays performed in Figure 3E. (B) Growth curves of PA14 in the presence of 100 µM compound. In panel A, error bars = standard deviations of biological replicates, $n = 2$, ns = $p > 0.05$ in two-tailed Student's t-test for each treatment compared to DMSO. In panel B, error bars = standard deviations of biological replicates, $n = 3$.

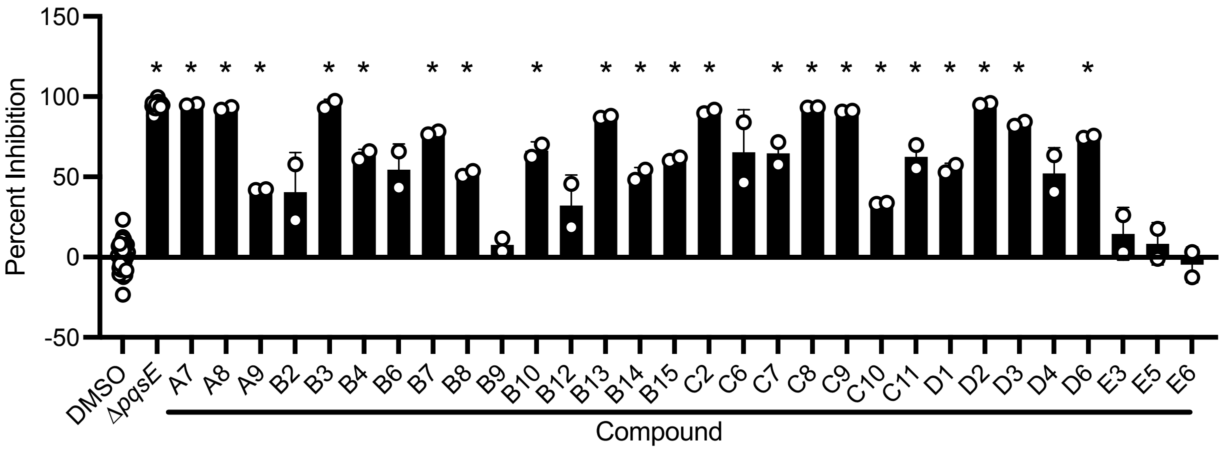

Figure S6. Inhibition of pyocyanin production in WT *P. aeruginosa* by 100 μM of the designated candidate compounds in the absence of SPR741. The left-most bar shows the result for DMSO as the negative control and the second bar shows the Δ*pqsE P. aeruginosa* mutant used as the positive control. Error bars = standard deviations of biological replicates, $n = 2$, * = $p < 0.05$ in two-tailed Student's t-test.

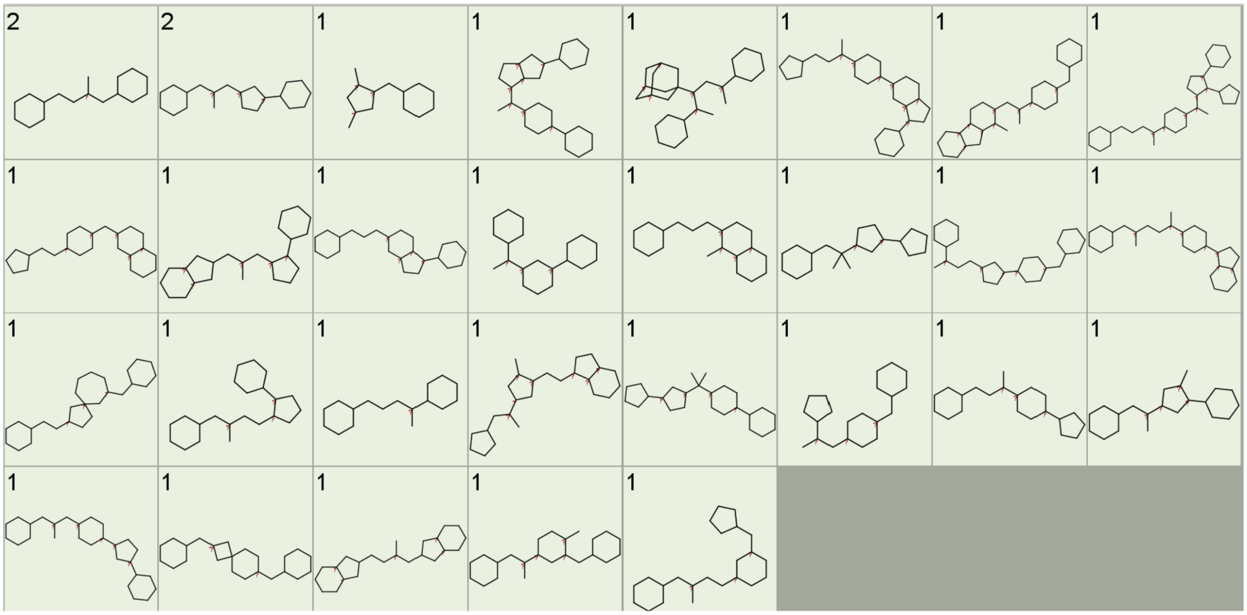

Figure S7. Structural diversity of PqsR inhibitor hits. Bemis-Murcko skeleton analysis of the hits indicated a range of chemical skeletons and a low frequency of duplicate skeletons. Bemis-Murcko skeletons[1] were calculated in DataWarrior (OpenChemLib v06.02.01)[2] and the frequency of each skeleton is shown in the top left corner of each structure box.

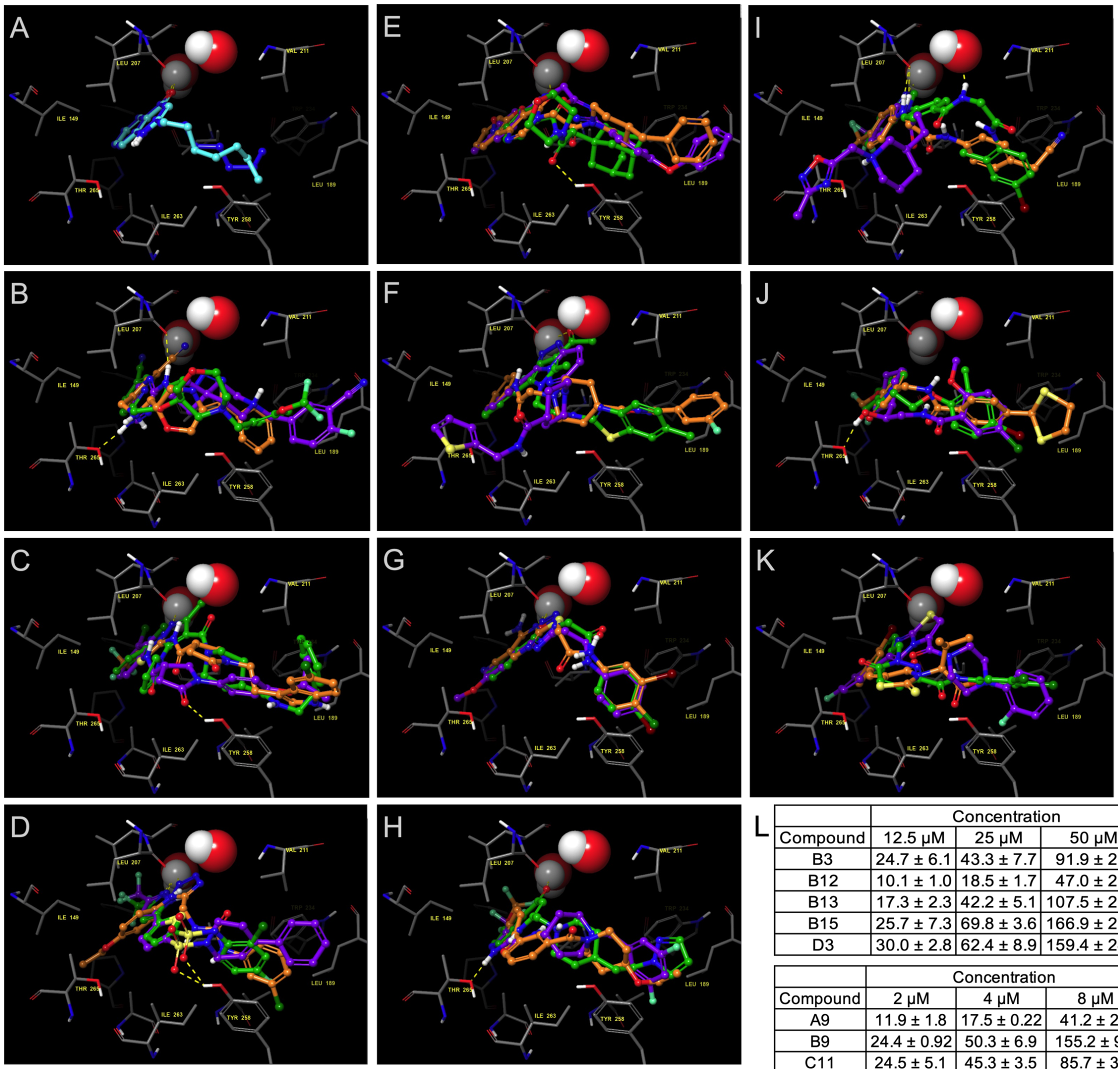

L

Compound	Concentration 12.5 μM	25 μM	50 μM	
B3	24.7 ± 6.1	43.3 ± 7.7	91.9 ± 2.7	EC_{50} (nM)
B12	10.1 ± 1.0	18.5 ± 1.7	47.0 ± 2.7	
B13	17.3 ± 2.3	42.2 ± 5.1	107.5 ± 25.0	
B15	25.7 ± 7.3	69.8 ± 3.6	166.9 ± 28.5	
D3	30.0 ± 2.8	62.4 ± 8.9	159.4 ± 24.2	

Compound	Concentration 2 μM	4 μM	8 μM	
A9	11.9 ± 1.8	17.5 ± 0.22	41.2 ± 2.2	EC_{50} (nM)
B9	24.4 ± 0.92	50.3 ± 6.9	155.2 ± 9.1	
C11	24.5 ± 5.1	45.3 ± 3.5	85.7 ± 3.6	
D2	17.7 ± 3.6	35.1 ± 3.0	109.0 ± 33.0	
D4	3.39 ± 0.67	9.32 ± 1.6	19.1 ± 3.0	

Compound	Concentration 0.5 μM	1 μM	2 μM	
A7	26.1 ± 5.1	56.1 ± 12.6	91.3 ± 11.0	EC_{50} (nM)
C7	19.0 ± 1.6	41.1 ± 1.9	79.9 ± 7.4	
C8	40.5 ± 8.0	83.1 ± 3.5	185.8 ± 21.3	
C9	27.8 ± 2.3	77.3 ± 6.4	130.8 ± 15.4	
D6	7.38 ± 0.82	17.0 ± 0.83	30.2 ± 10.4	

Figure S8. (A) Overlay of the HHQ ligand determined in the PqsR x-ray structure 6Q7U[3] (light blue) and docked into the same protein structure using our docking method (dark blue). (B-K) PqsR inhibitors identified in the current work docked into PqsR x-ray structure 6Q7U. In each panel, water molecules are shown in CPK representation and atoms are colored as follows: oxygen (red), nitrogen (dark blue), hydrogen (white, only hydrogen atoms attached to heteroatoms are shown), sulfur (yellow), chlorine (dark green), fluorine (light green), carbons of PqsR protein (gray). Carbon atoms of docked inhibitor compounds are colored as follows: (B) A7 (purple), A8 (green), A9 (orange); (C) B2 (purple), B3 (green), B4 (orange); (D) B6 (purple), B7 (green), B8 (orange); (E) B9 (purple), B10 (green), B12 (orange); (F) B13 (purple), B14 (green), B15 (orange); (G) C2 (purple), C6 (green), C7 (orange); (H) C8 (purple), C9 (green), C10 (orange); (I) C11 (purple), D1 (green), D2 (orange); (J) D3 (purple), D4 (green), D6 (orange); (K) E3 (purple), E5 (green), E6 (orange). A yellow dashed line indicates a hydrogen bond. (L) EC_{50} values (nM) from competition assays with inhibitors against PQS. These data are presented in Figure 4C-E as values relative to the EC_{50} resulting from addition of the lowest concentration of the given inhibitor.

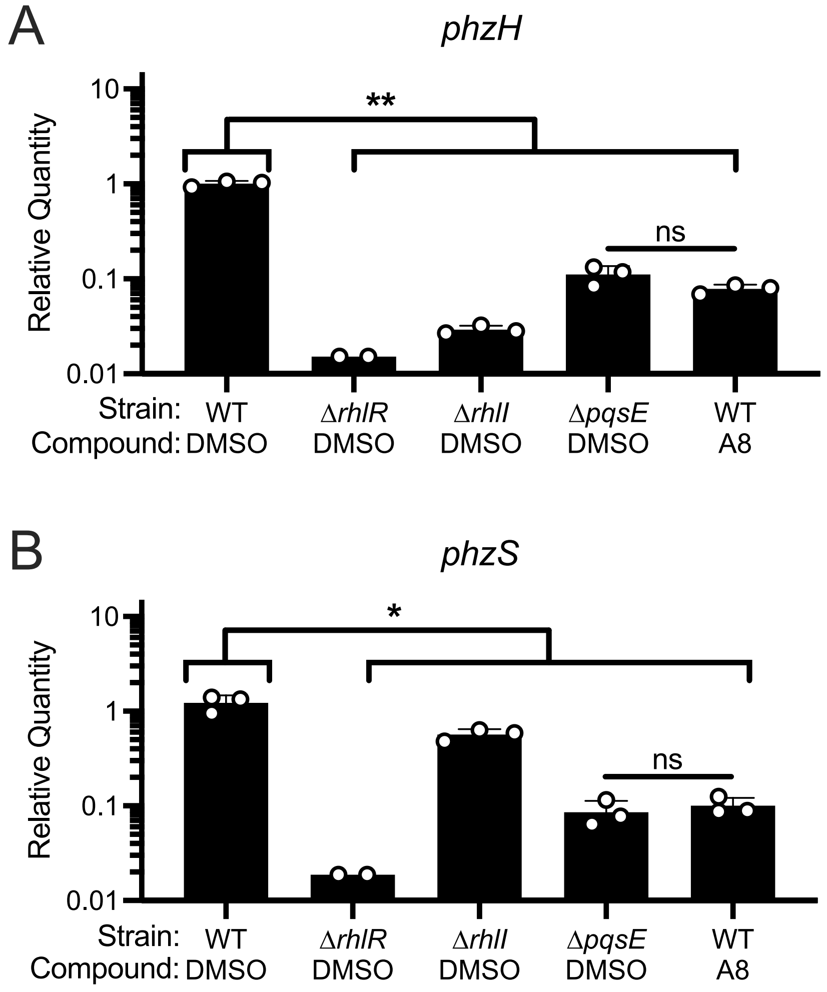

Figure S9. Quantitative PCR measurements of *phzH* (A) and *phzS* (B) in the designated strains in the presence of 1% (v/v) DMSO or the PqsR inhibitor A8 at 100 µM. Error bars = standard deviations of biological replicates, $n = 3$, * = $p < 0.05$, ** = $p < 0.005$, and ns = $p > 0.05$ in two-tailed Student's t-test.

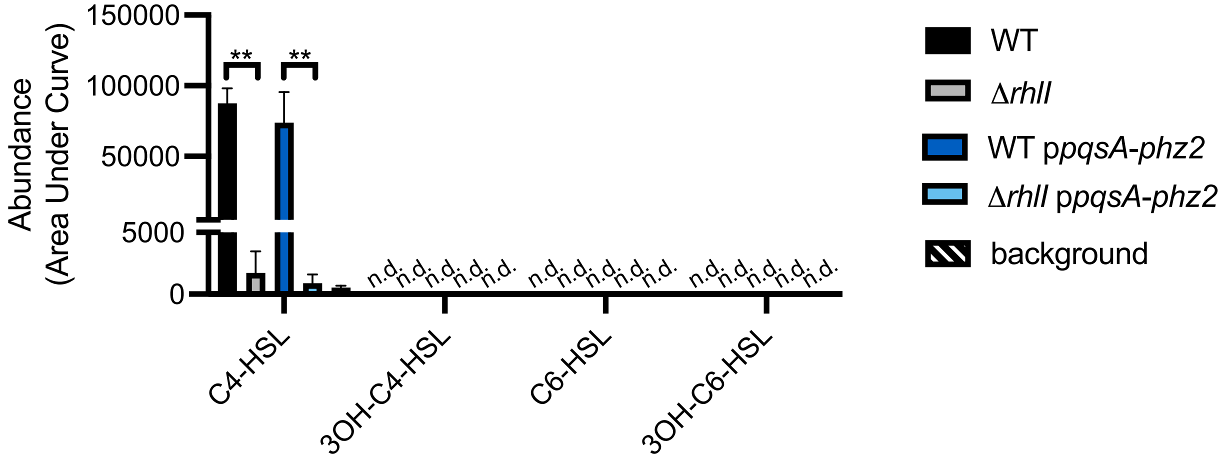

Figure S10. Quantitation of designated HSLs in WT and Δ*rhlI P. aeruginosa* with and without p*pqsA-phz2*. Error bars = standard deviations of biological replicates, *n* = 3, * = p < 0.05 and ** = p < 0.005 in two-tailed Student's t-test. The *P. aeruginosa* Δ*rhlI* strains (with and without the p*pqsA-phz2* modification) showed no significant difference compared to the background.

Table S1. Docking analysis of PqsR inhibitors

Compound	Structure	Glide GScore (kcal/mol)	Tot Q (kcal/mol)	State Penalty
A7	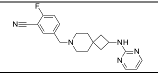	-6.8	1	0.01
A8	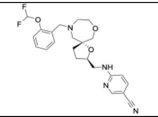	-7.5	1	0.30
A9	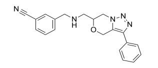	-7.4	1	0.33
B2	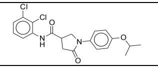	-7	0	0.00
B3	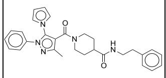	-6.5	0	0.00
B4	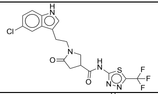	-7.1	0	0.37
B6	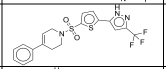	-6.3	0	0.30
B7	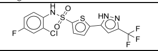	-6.7	-1	0.41
B8	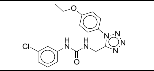	-6.9	0	0.00
B9	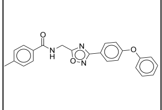	-7.1	0	0.00
B10	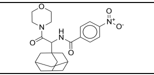	-5.8	0	0.00
B12	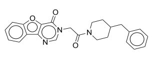	-6.1	0	0.00
B13	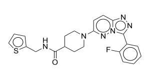	-6.4	0	0.00
B14	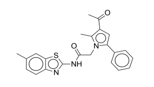	-6.6	-1	0.81
B15	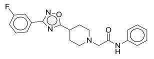	-7.2	1	0.47
C2	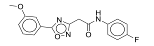	-7.9	0	0.00
C6	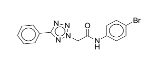	-7.5	0	0.00
C7	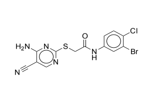	-6.5	0	0.00
C8	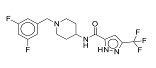	-8.4	0	0.49
C9	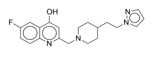	-8	1	0.14
C10	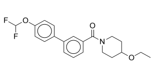	-5.9	0	0.00
C11	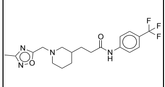	-7.8	1	0.00
D1	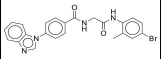	-7.3	0	0.01
D2	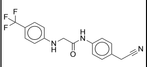	-7.7	0	0.00
D3	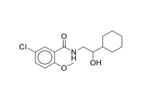	-6.2	0	0.00
D4	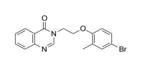	-7.5	0	0.00
D6	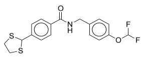	-6.7	0	0.00
E3	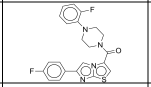	-7	0	0.00
E5	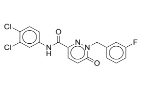	-6.8	0	0.00
E6	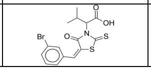	-5.7	-1	0.00

Footnote: Structures of docked PqsR inhibitors with their best docking Glide GScores (kcal/mol), total charge of docked ligand (Tot Q), and state penalty (kcal/mol). Glide GScore is used to predict the binding affinity of a ligand to a target protein[61]. Lower GScores indicate potential for higher binding affinity.

Table S2 - Strain list

Strain	Genotype	Reference
JSV1310	PA14 p*phz1-lux*	This work
JSV1563	PA14 p*phz1-lux* Δ*lasR*	This work
JSV1561	PA14 p*phz1-lux* Δ*lasI*	This work
JSV1559	PA14 p*phz1-lux* Δ*rhlR*	This work
JSV1556	PA14 p*phz1-lux* Δ*rhlI*	This work
JSV1534	PA14 p*phz1-lux* Δ*pqsR*	This work
JSV1553	PA14 p*phz1-lux* Δ*pqsA*	This work
JSV1362	PA14 p*phz1-lux* Δ*pqsE*	This work
JSV1558	PA14 p*phz1-lux* Δ*rhlI* Δ*pqsE*	This work
JSV1402	PA14 p*phz1-lux pqsE*::*pqsE*-D73A	This work
JSV1397	PA14 p*phz1-lux pqsE*::*pqsE*-NI	This work
JSV1423	PA14 p*chiC-lux*	This work
JSV1568	PA14 p*chiC-lux* Δ*rhlR*	This work
JSV1538	PA14 p*chiC-lux* Δ*pqsR*	This work
JSV1566	PA14 p*chiC-lux* Δ*lasR*	This work
JSV1269	PA14 p*hcnA-lux*	This work
JSV1570	PA14 p*hcnA-lux* Δ*rhlR*	This work
JSV1536	PA14 p*hcnA-lux* Δ*pqsR*	This work
JSV1591	PA14 p*hcnA-lux* Δ*lasR*	This work
BB-Pa426	PA14 p*rhlA-lux*	This work
JSV1593	PA14 p*rhlA-lux* Δ*rhlR*	This work
JSV1587	PA14 p*rhlA-lux* Δ*pqsR*	This work
JSV1589	PA14 p*rhlA-lux* Δ*lasR*	This work
JSV1269	PA14 p*phz2-lux*	This work
JSV1578	PA14 p*phz2-lux* Δ*rhlR*	This work
JSV1533	PA14 p*phz2-lux* Δ*pqsR*	This work
JSV1586	PA14 p*phz2-lux* Δ*lasR*	This work
JSV1364	PA14 p*rpsL-lux*	This work
JSV1569	PA14 p*rpsL-lux* Δ*rhlR*	This work
JSV1530	PA14 p*rpsL-lux* Δ*pqsR*	This work
JSV1567	PA14 p*rpsL-lux* Δ*lasR*	This work
JSV1390	PA14 p*tac-lux*	This work
JSV1579	PA14 p*tac-lux* Δ*rhlR*	This work
JSV1542	PA14 p*tac-lux* Δ*pqsR*	This work
JSV1583	PA14 p*tac-lux* Δ*lasR*	This work
JSV1378	PA14 Tn*7*-p*phz1-lux*, *ect6*-p*lac-tomato*	This work
JSV1384	PA14 Tn*7*-p*phz1-lux*, *ect6*-p*lac-tomato* Δ*pqsE*	This work
PA14	WT *P. aeruginosa*	[4]
BB-Pa0482	PA14 Δ*pqsE*	[5]
BB-Pa0555	PA14 Δ*pqsR*	This work
JSV1552	TOP10 pCS26-p*pqsA-luxCDABE* pBAD-*pqsR*	This work
BB-Ec0386	TOP10 pCS26-p*rhlA-luxCDABE* pBAD-*rhlR*	[6]
BB-Ec0382	TOP10 pCS26-p*lasB-luxCDABE* pBAD-*lasR*	[6]

JSV1404	PA14 Tn*7*-p*tac-lux*, *ect6*-p*lac-tomato*	This work
JSV1629	PA14 p*pqsA-phz2*	This work
JSV1637	PA14 p*pqsA-phz2* Δ*pqsE*	This work
JSV1638	PA14 p*pqsA-phz2* Δ*rhlI*	This work
JSV1643	PA14 p*pqsA-phz2* Δ*rhlR*	This work
JSV1548	PA14 p*phz1-lux* pUCP18	This work
JSV1549	PA14 p*phz1-lux* pUCP18-p*lac-pqsE*	This work
JSV1544	PA14 p*phz1-lux* Δ*pqsR* pUCP18	This work
JSV1545	PA14 p*phz1-lux* Δ*pqsR* pUCP18-p*lac-pqsE*	This work

Table S3 - Primer list

Primer name	Primer Sequence	Primer Purpose
JSV1075	GCACATCGGCGACGTGCTCTC	Confirm reporter integration
JSV1076	CTGTGCGACTGCTGGAGCTGA	Confirm reporter integration
JSV1077	CACAGCATAACTGGACTGATTTC	Confirm reporter integration
JSV1078	ATTAGCTTACGACGCTACACCC	Confirm reporter integration
JSV1059	ATTTCATTCATTATTAACGGCCAGGTTG	Amplify vector for reporters
JSV1060	GGATCCACTAGTGAGCTCATGC	Amplify vector for reporters
JSV1063	CGATCATGCATGAGCTCACTAGTGGATCCTTCGATCAGCGTAGTGACG	Amplify p*hcnA*
JSV1064	CTGGCCGTTAATAATGAATGAAATCTTCTTAGTCATTGCCCTTTCATCCGTGAGAG	Amplify p*hcnA*
JSV1239	GAGCTCACTAGTGGATCGTGAACGGCGCG	Amplify p*chiC*
JSV1240	GAATGAAATCTTCTTAGTCATTTACCGCTCCTCGGATG	Amplify p*chiC*
PrhlA-lux F	CGATCATGCATGAGCTCACTAGTGGATCCGAAGATCTACGCCAACGAAGG	Amplify p*rhlA*
PrhlA-lux R	CTGGCCGTTAATAATGAATGAAATCTTCTTAGTCATCTCACACCTCCCAAAAATTTTC	Amplify p*rhlA*
JSV1067	CGATCATGCATGAGCTCACTAGTGGATCCAAGCGCTCTATTTCGCACTTCTTGCCC	Amplify p*phz1*
JSV1068	CTGGCCGTTAATAATGAATGAAATCTTCTTAGTCATGCGCCGCCTCCGAGAGGGCTC	Amplify p*phz1*
JSV1069	CGATCATGCATGAGCTCACTAGTGGATCCGCAAGCTCAACTCCAGCAACA	Amplify p*phz2*

JSV1070	CTGGCCGTTAATAATGAATGAAATCTTCTTAGTCATGGTGCGAATCTCCGCCAGTTC	Amplify p*phz2*
JSV1166	CGATCATGCATGAGCTCACTAGTGGATCAATCAACGACAAGCACATCG	Amplify p*rpsL*
JSV1167	CTGGCCGTTAATAATGAATGAAATCTTCTTAGTCATCTATAGCTCCACTGATTGTCTTACG	Amplify *rpsL* promoter
JSV1216	CATTCCACTTACAATTAGGCAAAGGATATGTAGGCTGGAGCTGCTTC	Amplify p*tac* promoter + luciferase
JSV1217	GCATGAGCTCACTAGTGGATCCTGCACCAATGCTTCTGGC	Amplify p*tac* promoter + luciferase
JSV1215	GAAGCAGCTCCAGCCTACATATCCTTTGCCTAATTGTAAGTGGAATG	Amplify vector for p*tac* reporter
JSV1218	GCCAGAAGCATTGGTGCAGGATCCACTAGTGAGCTCATGC	Amplify vector for p*tac* reporter
JSV1501	GGGAACGTTCTGTCATGCGAGAGTACCAACG	Replace p*phz2* with p*pqsA*
JSV1502	TGGTACTCTCGCATGACAGAACGTTCCCTCTTC	Replace p*phz2* with p*pqsA*
JSV1503	GGGGATCCTCTAGAGAATAATCATTAATCAGGTGGGAATG	Replace p*phz2* with p*pqsA*
JSV1504	CGTTTGCGCCGGTTCGCCCTTCTTGCTTGG	Replace p*phz2* with p*pqsA*
JSV1506	AAGCAAGAAGGGCGAACCGGCGCAAACG	Replace p*phz2* with p*pqsA*
JSV1508	ATTAATGATTATTCTCTAGAGGATCCCCGGG	Replace p*phz2* with p*pqsA*
JSV1509	TGTAAAGCAAGCTTGTTTCTTCTGGGAGGAAGC	Replace p*phz2* with p*pqsA*
JSV1509	CTCCCAGAAGAAACAAGCTTGCTTTACATTTATGCTTC	Replace p*phz2* with p*pqsA*
JSV1195	CAAAACTGCCCGCAACGT	*rpsL* quantitative PCR
JSV1196	TTTCGGCGTGGTGGTGTAT	*rpsL* quantitative PCR
JSV1551	GATCTTCGCCATGACCGATAC	*phzH* quantitative PCR
JSV1557	GTGGGAAAGCGATAGGACATC	*phzH* quantitative PCR
JSV1443	CGTCGGCATCAATATCCAGC	*phzS* quantitative PCR
JSV1444	CGTCGGCATCAATATCCAGC	*phzS* quantitative PCR
ES-D-57	cagcgaatcggatacgcaac	Amplify 5' upstream of PqsR - F
ES-D-58	ttcagcatgcttgcggctcgagttcatcccttattccttttattgggtgg	Amplify 5' upstream of PqsR - R
ES-D-59	aactcgagccgcaagcatgctgaatagagcgttctccagcagacg	Amplify 3' downstream of PqsR-F
ES-D-60	cagttgctgcggcagtacg	Amplify 3' downstream of PqsR-R

ES-D-61	ggaagcataaatgtaaagcagtcgctacacctgaaggcg	Amplify PqsR deletion construct for insertion into pEXG2
ES-D-62	gagcccggggatcctcaggcgccggcgt	Amplify PqsR deletion construct for insertion into pEXG2

Table S4. Analytical data for purchased compounds

Compound	Ion Type	Expected m/z	Observed m/z	ppm Error	Percent Purity
A07	[M+H]	352.1932	352.1959	-7.72	>80%
A08	[M+H]	445.2046	445.2073	-6.11	>95%
A09	[M+H]	346.1663	346.1679	-4.68	>95%
B10	[M+H]	428.2180	428.2199	-4.48	>95%
B12	[M+H]	402.1812	402.1832	-5.02	>95%
B13	[M+H]	437.1555	437.1586	-7.14	>95%
B14	[M-H]	402.1281	402.1289	-1.92	>95%
B15	[M+H]	381.1722	381.1742	-5.30	>95%
B02	[M-H]	405.0778	405.0790	-2.89	>95%
B03	[M+H]	482.2551	482.2572	-4.40	>95%
B04	[M+H]	458.0660	458.0679	-4.19	>95%
B06	[M+H]	440.0709	440.0700	2.00	>95%
B07	[M-H]	423.9610	423.9626	-3.71	>60%
B09	[M+H]	386.1499	386.1480	4.87	>95%
C10	[M+H]	376.1719	376.1739	-5.37	>95%
C11	[M+H]	397.1846	397.1866	-5.09	>95%
C02	[M+H]	328.1092	328.1111	-5.85	>95%
C06	[M-H]	356.0152	356.0161	-2.45	>95%
C07	[M+H]	397.9473	397.9487	-3.57	>95%
C08	[M+H]	389.1396	389.1420	-6.22	>95%
C09	[M+H]	355.1929	355.1945	-4.56	>95%
D01	[M+H]	463.0764	463.0784	-4.36	>95%
D02	[M+H]	334.1162	334.1179	-5.15	>95%
D03	[M+H]	312.1361	312.1369	-2.63	>95%
D04	[M+H]	359.0390	359.0392	-0.61	>95%
D06	[M+H]	382.0742	382.0758	-4.24	>95%
E03	[M+H]	425.1242	425.1258	-3.81	>95%
E05	[M+H]	392.0364	392.0385	-5.41	>95%
E06	[M-H]	397.9525	397.9535	-2.44	>95%

References

(1) Bemis, G. W.; Murcko, M. A. The Properties of Known Drugs. 1. Molecular Frameworks. *J. Med. Chem.* **1996**, *39* (15), 2887–2893. https://doi.org/10.1021/jm9602928.

(2) Sander, T.; Freyss, J.; von Korff, M.; Rufener, C. DataWarrior: An Open-Source Program for Chemistry Aware Data Visualization and Analysis. *J. Chem. Inf. Model.* **2015**, *55* (2), 460–473. https://doi.org/10.1021/ci500588j.

(3) Zender, M.; Witzgall, F.; Kiefer, A.; Kirsch, B.; Maurer, C. K.; Kany, A. M.; Xu, N.; Schmelz, S.; Börger, C.; Blankenfeldt, W.; Empting, M. Flexible Fragment Growing Boosts Potency of Quorum-Sensing Inhibitors against *Pseudomonas aeruginosa* Virulence. *ChemMedChem* **2020**, *15* (2), 188–194. https://doi.org/10.1002/cmdc.201900621.

(4) Lau, G. W.; Ran, H.; Kong, F.; Hassett, D. J.; Mavrodi, D. *Pseudomonas aeruginosa* Pyocyanin is Critical for Lung Infection in Mice. *Infect. Immun.* **2004**, *72* (7), 4275–4278. https://doi.org/10.1128/IAI.72.7.4275-4278.2004.

(5) Mukherjee, S.; Moustafa, D. A.; Stergioula, V.; Smith, C. D.; Goldberg, J. B.; Bassler, B. L. The PqsE and RhlR Proteins are an Autoinducer Synthase–Receptor Pair that Control Virulence and Biofilm Development in *Pseudomonas aeruginosa*. *Proc. Natl. Acad. Sci.* **2018**, *115* (40), E9411–E9418. https://doi.org/10.1073/pnas.1814023115.

(6) Paczkowski, J. E.; Mukherjee, S.; McCready, A. R.; Cong, J.-P.; Aquino, C. J.; Kim, H.; Henke, B. R.; Smith, C. D.; Bassler, B. L. Flavonoids Suppress *Pseudomonas aeruginosa* Virulence through Allosteric Inhibition of Quorum-Sensing Receptors. *J. Biol. Chem.* **2017**, *292* (10), 4064–4076. https://doi.org/10.1074/jbc.M116.770552.
